# Functional Changes Associated With the Subcellular Localization of the Nuclear Receptor NR4A1

**DOI:** 10.1155/bri/4849733

**Published:** 2025-07-24

**Authors:** Yoshimitsu Kiriyama, Akira Nakatsuma, Hiroshi Tokumaru, Hisayo Sadamoto, Hiromi Nochi

**Affiliations:** ^1^Kagawa School of Pharmaceutical Sciences, Tokushima Bunri University, Takamatsu 769-2193, Kagawa, Japan; ^2^Institute of Neuroscience, Tokushima Bunri University, Takamatsu 769-2193, Kagawa, Japan

**Keywords:** apoptosis, autophagy, mitophagy, NGFI-B, NR4A1, NUR77, TR3

## Abstract

Nuclear receptor subfamily 4 group A member 1 (NR4A1), which is also known as nuclear receptor 77 (NUR77), NGFI-B, or testicular receptor 3 (TR3), is a member of the NR4A subfamily of the nuclear receptor superfamily. NR4A1 has both nuclear localization and nuclear export signals, and NR4A1 is present in the cytoplasm as well as the nucleus. NR4A1 alters its subcellular localization through phosphorylation or SUMOylation. In the nucleus, NR4A1 plays an important role in regulating gene expression by directly binding to genomic DNA or indirectly influencing other transcription factors. In the cytoplasm, NR4A1 affects the stabilization of β-catenin, which is involved in various tumorigenesis. Furthermore, NR4A1 is involved in LPS-induced inflammasome activation. In mitochondria and endoplasmic reticulum, NR4A1 plays an important role in the induction of autophagy and apoptosis. In this review, we focused on the current knowledge of subcellular localization and molecular function of NR4A1.

## 1. Introduction

### 1.1. The Nuclear Receptor Superfamily

Nuclear receptors have the DNA-binding domain (DBD) to bind genomic DNA and the ligand-binding domain (LBD) to bind their unique ligands. Nuclear receptors form a family called the nuclear receptor superfamily because of their similar amino acid sequences and domain structures. In humans, the nuclear receptor superfamily consists of 48 members. Based on molecular phylogeny, nuclear receptors can be classified into seven subfamilies: nuclear receptor 1 (NR1), NR2, NR3, NR4, NR5, NR6, and NR0 [1, 2]. The NR1 subfamily consists of thyroid hormone receptor-like nuclear receptors. The NR2 subfamily consists of retinoid X receptor (RXR)-like nuclear receptors. The NR3 subfamily consists of estrogen receptor-like nuclear receptors. The NR4 subfamily consists of nerve growth factor (NGF)-induced B (NGFI-B)-like nuclear receptors. The NR5 subfamily consists of steroidogenic factor 1-like nuclear receptors. NR6 family consists of germ cell nuclear factor, also known as RXR-related testis-associated receptor. The NR0 subfamily consists of atypical nuclear receptors, which are short heterodimeric partner (SHP) and dosage-sensitive sex reversal-adrenal hypoplasia congenital critical region on the X chromosome, gene 1 (DAX1). SHP and DAX1 lack the DBD region in their structures and function as transcriptional regulators by binding to other nuclear receptors [1, 3]. The activities of most nuclear receptor superfamily members are regulated by small lipophilic molecules as ligands. However, despite having the basic structure of nuclear receptors, ligands for several members of the nuclear receptor superfamily have not been identified, and these members of the nuclear receptor superfamily are referred to as orphan nuclear receptors [1, 4].

### 1.2. Nuclear Receptor Subfamily 4 Group A Member 1 (NR4A1)

**Figure 1:**
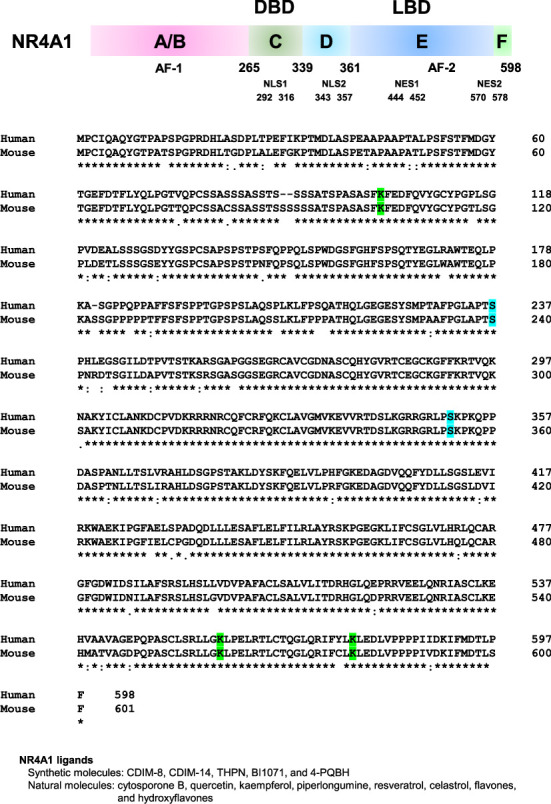
General features, amino acid sequence, and ligands of human NR4A1. C and E indicate DNA-binding domain (DBD) and ligand-binding domain (LBD), respectively. Human and mouse NR4A1 proteins were aligned using Clustal Omega. Amino acid residues fully conserved in human and mouse sequences are indicated by asterisks (∗) in the bottom line. Colon (:) indicates highly similar properties, and a period (.) indicates less similar properties. Phosphorylation sites involved in the subcellular distribution of NR4A1 are highlighted in blue, and SUMOylation sites of NR4A1 are highlighted in green. C. Synthetic and natural ligands of NR4A1. Abbreviations: AF-1 = Activator function-1, AF-1 = Activator function-2, BI1071 = Di(1H-indol-3-yl) (4-(trifluoromethyl)phenyl)methane, CDIM-8 = 1,1-bis(3′-indolyl)-1-(p-hydroxyphenyl)methane, CDIM-14 = 1,1-bis(3′-indolyl)-1-1(p-carbomethoxyphenyl)methane, NES = Nuclear export signal, NLS = Nuclear localization signal, THPN = 1-(3,4,5)-trihydroxyphenyl)nonan-1-one, 4-PQBH = 4-(quinoline-4-amino)benzoylhydrazide.

NR4A1, which is also known as nuclear receptor 77, NGFI-B, and testicular receptor 3, is one of the orphan receptors and a member of the NR4A subfamily (Figure 1). The NR4A subfamily consists of NR4A1, NR4A2 (also known as Nur-related factor 1), and NR4A3 (also known as neuron-derived orphan receptor 1). NR4A1 was first identified as a protein that was induced by serum in mouse fibroblasts and by NGF in the rat adrenal pheochromocytoma cell line PC12 [5, 6]. Human NR4A1 was isolated from a human prostate λgt11 cDNA library using oligonucleotide probes to the DBD common to the nuclear receptor superfamily [7]. NR4A1 is widely expressed in various tissues [8] and plays a crucial role in the regulation of cell growth, apoptosis, the immune system, and metastasis in various tumors, such as lung and liver cancers and leukemia [9–12]. Although NR4A1 is one of the orphan nuclear receptors, cytosporone B, which is isolated from *Cytospora* sp., and several small synthetic molecules act as ligands for NR4A1 [13–15]. Furthermore, plant-derived small molecules, such as quercetin, kaempferol, piperlongumine, and resveratrol, bind NR4A1 and inhibit the transcription activity of NR4A1 [16–19]. Flavone and hydroxyflavones, such as 7-hydroxyflavone and 3,4-dihydroxyflavone, bind NR4A1 and promote the transcription activity of NR4A1 [20]. In addition, it has been reported that unsaturated fatty acids, such as palmitoleic acid and arachidonic acid, can bind to NR4A1 [21]. Although NR4A1 is one of the nuclear receptors and directly regulates gene expression, NR4A1 also functions outside the nucleus without directly influencing gene expression. This review focused on the current knowledge of subcellular localization and molecular function of NR4A1.

## 2. Structure and Posttranslational Modification of NR4A1

### 2.1. Structure of NR4A1

The basic structure of nuclear receptors has different functional domains: N-terminal domain, DBD, hinge, and LBD. The N-terminal domain contains an activator function-1 region (also called the A/B region). Activator function-1 varies in size and is highly diverse across nuclear receptors. Activator function-1 interacts with transcriptional coregulatory proteins, such as coactivators or corepressors, and is associated with ligand-independent transcriptional activation. The DBD (also called the C region) is a domain that binds to genomic DNA and is located next to the N-terminal domain, placing it at the center of the nuclear receptor. The DBD is a highly conserved region among nuclear receptors. The hinge region (also called the D region) links the DBD and LBD of a nuclear receptor and is the most diverse in sequence and size among nuclear receptors. The LBD (also called the E region) can bind to lipophilic small molecules, such as steroid derivatives, retinoids, and fatty acids. The LBD contains an activator function-2 region and interacts with transcriptional coregulatory proteins [1]. The members of the NR4A subfamily are highly homologous in the DBD (91%–95%) and moderately homologous in the LBD (∼60%) but are divergent in the N-terminal A/B region [22]. NR4A1 has nuclear localization signals (NLSs) and nuclear export signals (NESs), and NR4A1 is present in the cytoplasm as well as the nucleus [23] (Figure 1). Protein transport between the cytoplasm and nucleus is mediated by importins and exportins, which recognize NLSs or NESs on target proteins [24]. NLSs are recognized by importin α. Importin α dimerizes with importin β, which mediates the interaction of target proteins with nuclear pores to transport target proteins into the nucleus [25]. Thus, the NLSs of NR4A1 and importins appear to play important roles in mediating the translocation of NR4A1 to the nucleus, whereas exportin 1, also known as chromosome region maintenance 1, plays an important role in transporting NR4A1 from the nucleus to the cytoplasm [26, 27].

### 2.2. Posttranslational Modification and Subcellular Localization of NR4A1

Phosphorylation of NR4A1 by various kinases induces the nuclear export of NR4A1. Phosphorylation of NR4A1 by Akt, c-Jun N-terminal kinase (JNK), and p90 ribosomal protein S6 kinase 2 induces NR4A1 translocation from the nucleus to the cytoplasm [28–33]. Akt phosphorylates NR4A1 at serine 351 and promotes NR4A1 translocation from the nucleus to the cytoplasm in 293, NIH 3T3, H460 lung cancer cells [28, 33]. NR4A1 is also phosphorylated by p90 ribosomal protein S6 kinase 2 at serine 351 in CD4^+^ CD8^+^ DO11.10 T-cell hybridoma. Moreover, mutation of NR4A1 to alanine, which blocks phosphorylation of serine 354, inhibited export from the nucleus, whereas mutation of NR4A1 to glutamate, which mimics phosphorylation of serine 354, promoted export from the nucleus [32]. Protein kinase C phosphorylates NR4A1 and induces NR4A1 translocation from the nucleus to mitochondria. However, a mutant in which serine 351 in NR4A1 is substituted with alanine has been shown to constitutively translocate NR4A1 to the mitochondrial/cytoplasmic fraction in 16610D9 CD4^+^ CD8^+^ cells. Thus, translocation of NR4A1 from the nucleus to the mitochondrial/cytoplasmic regions may be cell type dependent [34]. ERK2 phosphorylates NR4A1 at serine 237 and facilitates NR4A1 translocation to mitochondria [35].

NR4A1 is SUMOylated at lysine 102, lysine 558, and lysine 577 [36, 37], and SUMOylation of NR4A1 leads to the translocation of NR4A1 to the cytoplasm from the nucleus [36]. Furthermore, SUMOylation inhibits the transcriptional activity of NR4A1 [36–38].

## 3. Regulation of Gene Expression by NR4A1 in the Nucleus

### 3.1. NR4A1 Response Elements

**Figure 2:**
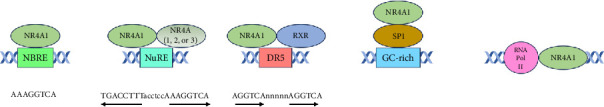
Interactions of NR4A1 with genomic DNA in the nucleus. NR4A1 regulates its target gene expression through binding to specific sites of genomic DNA. NR4A1 binds as a monomer to the NGFI-B response element (NBRE). NR4A1 forms homodimer or heterodimer with NR4A2 or NR4A3 and binds to the Nur-response element (NurRE) and forms a heterodimer with RXR to bind to direct repeat 5 (DR5). Furthermore, NR4A1 indirectly binds to GC-rich promoter regions (GC-rich) via SP-1/4. NR4A1 also binds to genomic DNA and prevents the RNA polymerase II transcriptional elongation.

The DBD of NR4A1 binds to the specific DNA sequence to regulate the expression of its target genes (Figure 2, Table 1). The sequences recognized by NR4A1 are (1) NGFI-B response element (NBRE) consisting of the AAAGGTCA sequence [39], (2) Nur-responsive element consisting of the everted repeat of the NBRE-related sequence separated by six nucleotides (TGATATTTacctccAAATGCCA) found in the pro-opiomelanocortin gene promoter and a consensus Nur-responsive element consisting of the everted repeat of perfect consensus half-sites identical to the NBRE (TGACCTTTacctccAAAGGTCA) [40, 41], and (3) direct repeat 5, which is a direct repeat of consensus hexamer half-site sequence (AGGTCA) separated by five nucleotide [42]. Monomer of NR4A1 binds to NBRE [39], and the homodimer of NR4A1 or heterodimer of NR4A1 with NR4A2 or NR4A3 binds to Nur-responsive element [40]. The heterodimer of NR4A1 with RXR binds to direct repeat 5 [42, 43].

### 3.2. Regulation of Gene Expression by NR4A1 Through Interaction With Several Transcription Factors and RNA Polymerase II

NR4A1 interacts with several transcription factors and regulates their activity. NR4A1 binds to specificity protein 1 (SP1) and SP4 [44]. SP1 and SP4 belong to the specificity proteins and Krüppel-like factor family and bind to GC-rich sequences [45]. The NR4A1 antagonist, such as 1,1-bis(3′-indolyl)-1-(p-hydroxyphenyl)methane (also known as CDIM-8) or 1,1-bis(3′-indolyl)-1-1(p-carbomethoxyphenyl)methane (also known as CDIM-14), reduces the transcriptional activity of SP1 and SP4 [44, 46–48]. NR4A1 also binds to gene bodies and 3′-untranslated regions of immediate-early genes, such as FOS, FOSB, and JUN, to inhibit the expression of these genes under serum-replete culture conditions in the human breast epithelial cell line MCF-10A. NR4A1 binds to RNA polymerase II to prevent the transcription of these immediate-early genes under serum-replete culture conditions. However, NR4A1 is released from immediate-early gene bodies under serum stress evoked by serum starvation followed by serum replenishment, and this leads to the induction of the expression of immediate-early genes [49]. However, the detailed mechanism by which NR4A1 binds to RNA polymerase II and the IEG gene body remains unknown and is expected to be elucidated, whereas phosphorylation of NR4A1 by Akt plays a role in the dissociation of NR4A1 from immediate-early gene bodies [49] because Akt phosphorylates NR4A1 and induces NR4A1 translocation from the nucleus to the cytoplasm [28]. NR4A1 associates with the p65 subunit of nuclear factor-κB and blocks nuclear factor-κB binding to a nuclear factor-κB response element [50]. NR4A1 binds to the activator protein 1 binding site and prevents activator protein 1-induced gene expression by blocking transcription by activator protein 1 [9]. In addition, peroxisome proliferator-activated receptor-γ binds to NR4A1, leading to the recruitment of tripartite motif 13, also known as ret finger protein 2, in the human breast cancer cell line MCF-7 [51]. Tripartite motif 13 is an endoplasmic reticulum (ER) transmembrane-anchored E3 ligase [52] and induces NR4A1 ubiquitination, leading to NR4A1 degradation [51]. NR4A1 also binds to p53, and the interaction of NR4A1 with p53 inhibits the binding of mouse double minute protein 2 (human ortholog is human double minute protein 2) to p53 [53]. p53 induces the expression of mouse double minute protein 2, which functions as an E3 ubiquitin ligase, and mouse double minute protein 2 can degrade p53 [54]. Thus, NR4A1 prevents p53 degradation by inhibiting mouse double minute protein 2 expression induced by p53 [55, 56]. Furthermore, the association between NR4A1 and p53 leads to the inhibition of the transcriptional activity of p53 [53]. DNA-dependent protein kinase (DNA-PK) is a serine/threonine protein kinase complex composed of DNA-PK catalytic subunit (DNA-PKcs) and Ku70/Ku80 heterodimer [57]. NR4A1 interacts with KU80 and inhibits the binding between KU80 and DNA. Furthermore, NR4A1 also interacts with DNA-PKcs, although this interaction is not dependent on KU80. DNA-PK can repair DNA double-strand breaks induced by ionizing radiation. However, NR4A1 binds to KU80 and prevents repair of DNA double-strand breaks, leading to apoptosis in human hepatoblastoma cell line HepG2. Ionizing radiation promotes NR4A1 phosphorylation by DNA-PKcs, leading to the phosphorylation and transcriptional activation of p53 [58]. The chromodomain helicase DNA-binding protein 1-like, also known as amplified in liver cancer 1, primarily regulates the maintenance of chromosome integrity and DNA repair by binding to DNA. The chromodomain helicase DNA-binding protein 1-like is also crucial as a transcriptional and translational activator of various genes [59]. The chromodomain helicase DNA-binding protein 1-like binds to NR4A1 and prevents NR4A1 translocation from the nucleus to mitochondria. Furthermore, the interaction between the chromodomain helicase DNA-binding protein 1-like and NR4A1 blocks staurosporine-induced apoptosis in hepatocellular carcinoma cells [60].

## 4. The Role of NR4A1 in the Cytoplasm

**Figure 3:**
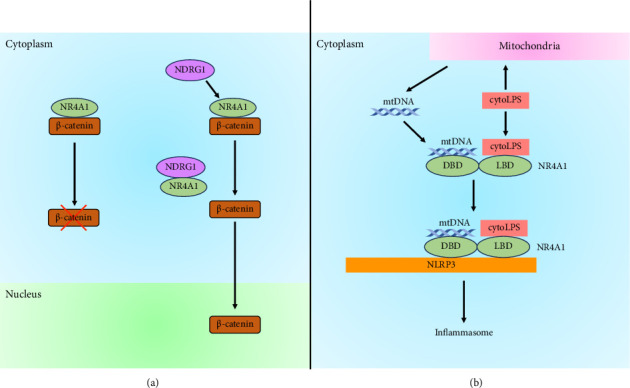
The role of NR4A1 in the cytoplasm. (a) NR4A1 interacts with β-catenin and is involved in β-catenin degradation in the cytoplasm, resulting in inhibition of β-catenin accumulation in the nucleus and tumor growth. N-myc downstream-regulated gene 1 (NDRG1) competitively binds to NR4A1, leading to the inhibition of β-catenin degradation. β-catenin accumulates in the nucleus and promotes tumor growth. (b) NR4A1 activates NLRP3 inflammasome by binding cytoplasmic LPS (cytoLPS) from infected bacteria and mitochondrial DNA (mtDNA) released from mitochondria. mtDNA is released by gasdermin D pore formation evoked by cytoLPS. cytoLPS and mtDNA bind to the LBD and DBD of NR4A1, respectively. This leads to the association between NR4A1 and NLR family pyrin domain containing 3 (NLRP), resulting in the inflammasome formation.

NR4A1 interacts with β-catenin and is involved in β-catenin degradation in the cytoplasm [26]. β-catenin plays an important role in the intracellular adhesion regulator and transcriptional activator, and stabilization of β-catenin has been significantly implicated in various cancers [61, 62]. Hellebritoxin H-9 from the skin of green toads [63] induces β-catenin degradation, which depends on NR4A1 and inhibits tumor growth [26]. In contrast, N-myc downstream-regulated gene 1 competitively binds to NR4A1 and disrupts the interaction between NR4A1 and β-catenin, leading to the inhibition of β-catenin degradation and β-catenin accumulation in the nucleus [64] (Figure 3(a)).

A recent report has shown that NR4A1 is a LPS-binding partner via the lipid A moiety of LPS [65] (Figure 3(b)). The stimulation of LPS (a Toll-like receptor 4 [TLR4] agonist), polyinosinic:polycytidylic acid (a synthetic analog of double-stranded RNA and TLR3 agonist), and Pam3CysSerLys4 (a synthetic triacylated lipopeptide and TLR1 and TLR2 agonist) induces NR4A1 expression in murine and human macrophages [65–67]. NR4A1 knockout in murine macrophages leads to the inhibition of noncanonical, but not canonical, inflammasome activation [65]. The activation of inflammasomes is classified into two pathways: canonical and noncanonical inflammasome activation. The canonical pathway is activated by two signals. The first signal is stimulated by recognizing extracellular pathogen-associated molecular patterns and danger-associated molecular patterns by pattern recognition receptors, such as TLR, nucleotide-binding oligomerization domain-like receptors, and C-type lectin receptors. This activates nuclear factor-κB and IRF3, leading to the expression of inflammasome sensors, procaspase-1, caspase-11, and interferon. Inflammasome sensors are absent in melanoma 2 inflammasome and the nucleotide-binding domain and leucine-rich repeat-containing (NLR) family inflammasomes. The second signal initiates inflammasome formation with an inflammasome sensor. NLR family inflammasomes include NLR family pyrin domain containing 1 (NLRP1), NLRP3, NLRP6, NLRP12, and NLR family caspase activation and recruitment domain containing 4. In contrast, the noncanonical pathway is activated via LPS-stimulated activation of caspases 4 and 5 in humans (the mouse ortholog is caspase 11), leading to NLRP3 activation, a cytoplasmic innate immune sensor of infection and inflammation [68]. Caspase-11 cleaves gasdermin D, and the released N-terminus of gasdermin D evokes pore formation in the plasma and mitochondrial membranes [69–72]. NR4A1 can interact with NLRP3 via the DBD in NR4A1. The double-stranded DNA (NBRE) binding to the DBD and cytoplasmic LPS binding to the LBD in NR4A1 are necessary for NR4A1 to bind NLRP3 to activate NLRP3 inflammasome. Furthermore, mtDNA is released by gasdermin D pore formation [73] and NBRE is present in mtDNA [65]. Thus, NR4A1 activates NLRP3 inflammasome by binding cytoplasmic LPS from infected bacteria and mtDNA released from mitochondria by gasdermin D activation.

### 4.1. Effects of NR4A1 on Autophagy and Apoptosis in Mitochondria

**Figure 4:**
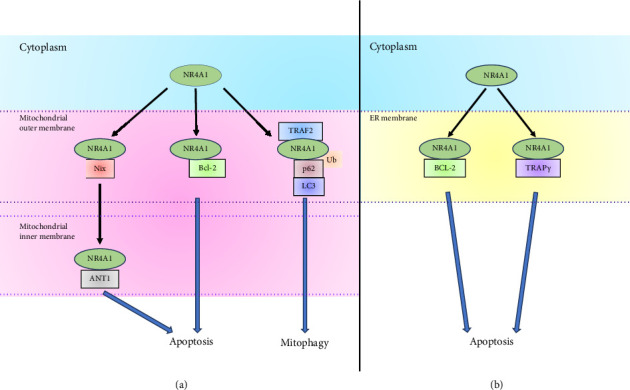
The role of NR4A1 in mitochondria and the ER. (a) In the mitochondria, NR4A1 can interact with proteins located in mitochondria; Nix, B-cell/chronic lymphocytic leukemia lymphoma 2 (Bcl-2), and TNF receptor-associated factor 2 (TRAF2). Association between NR4A1 and Nix induces the translocation of NR4A in the mitochondrial outer membrane and adenine nucleotide translocator-1 (ANT1), leading to apoptosis. Association between NR4A1 and Bcl-2 in the mitochondrial outer membrane induces the exposure of Bcl-2 homology 3 domain of Bcl-2, leading to apoptosis. Association between NR4A1 and tumor necrosis factor receptor-associated factor 2 (TRAF2) induces NR4A1 ubiquitination. p62 recruits to ubiquitinated NR4A1, leading to the association of LC3 to induce mitophagy in the mitochondrial outer membrane. (b) In the endoplasmic reticulum (ER), the association between NR4A1 and Bcl-2 or translocon-associated protein subunit γ (TRAPγ) in the ER membrane induces apoptosis.

NR4A1 can localize to mitochondria by interacting with several proteins located in mitochondria, such as Nix (also called Bcl-2/adenovirus E1B 19 kDa protein-interacting protein 3-like), B cell/chronic lymphocytic leukemia lymphoma 2 (Bcl-2), and tumor necrosis factor receptor-associated factor 2 (Figure 4). NR4A1 is translocated from the cytoplasm to mitochondria by treatment of 1-(3,4,5)-trihydroxyphenyl)nonan-1-one (THPN), a synthetic NR4A1 ligand, and NR4A1 treated with THPN leads to a decrease in the mitochondrial membrane potential and an induction of autophagic cell death in human melanoma cell line A375 [74]. NR4A1 treated with THPN interacts with Nix located in the mitochondrial outer membrane (MOM). Nix is located in the MOM and associates with LC3 to form phagosomes in mitophagy [75, 76]. NR4A1 that reaches the mitochondria reduces mitochondrial membrane potential by binding to the adenine nucleotide translocator-1, which is located in the mitochondrial inner membrane (MIM) and forms the channel of adenosine triphosphate and adenosine diphosphate [74]. The translocation of NR4A1 from the MOM to MIM is conducted by passing through the translocase of the outer mitochondrial membrane (TOM) complex from the MOM [74]. The TOM complex is the main entry gate for the import of mitochondrial proteins and is located in the MOM. The TOM complex consists of seven subunits: Tom40, a β-barrel membrane protein that constitutes the channel, and Tom20, Tom22, Tom70, Tom5, Tom6, and Tom7, which are single-transmembrane membrane proteins with an α-helix structure [77, 78]. Tom40 and Tom70 are necessary for NR4A1 translocation to the MIM [74]. Glucose deprivation increases reactive oxygen species levels and induces cell death. NR4A1 in the cytoplasm is phosphorylated by ERK2, which is activated by reactive oxygen species. In turn, phosphorylation of NR4A1 leads to translocation of NR4A1 from the cytoplasm to mitochondria by interacting with Nix [35]. Moreover, NR4A1 interacts with TPβ in mitochondria via the LBD of NR4A1. TPβ is the rate-limiting enzyme during fatty acid oxidation but is also associated with a reduction of reactive oxygen species levels and cell death. Glucose deprivation induces oxidation of TPβ, leading to the inhibition of TPβ activity. However, NR4A1 prevents TPβ oxidation by oxidizing NR4A1 itself, thereby keeping TPβ active. This leads to the prevention of glucose deprivation-induced cell death of melanoma cells [35].

The LBD of NR4A1 can bind to the N-terminal loop region of Bcl-2. Bcl-2 belongs to the Bcl-2 family, and members of the Bcl-2 family have BH domains and are involved in apoptosis. The association of NR4A1 with Bcl-2 leads to NR4A1 translocation to mitochondria, apoptosis, and the conformation change of Bcl-2 to expose its Bcl-2 homology 3 domain and functional change of Bcl-2 from antiapoptotic to proapoptotic [79, 80]. NR4A1-derived peptide with nine amino acids also binds to the loop region of Bcl-2 and exposes the Bcl-2 homology 3 domain, leading to apoptosis in mouse embryonic fibroblasts [81]. Di(1H-indol-3-yl) (4-(trifluoromethyl)phenyl)methane (BI1071) binds to NR4A1 and induces the association between NR4A1 and Bcl-2, leading to NR4A1 translocation to mitochondria and apoptosis induction in mouse embryonic fibroblasts and human cervical carcinoma cell line HeLa [82]. Activation of Ras-related C3 botulinum toxin substrate 1, which is a small GTPase, leads to the nuclear localization of T-cell lymphoma invasion and metastasis-inducing protein 1. T-cell lymphoma invasion and metastasis-inducing protein 1 interacts with NR4A1 to keep NR4A1 in the nucleus, inhibiting Bcl-2 homology 3 exposure in Bcl-2 by NR4A1 [83]. Bcl-2 family protein resembling Boo and BFL-1 are prosurvival Bcl-2 paralog and members of the Bcl-2 family and expressed in mitochondria [84–87]. NR4A1 can also bind to Bcl-B and BFL-1, and NR4A1 interaction with Bcl-B or BFL-1 leads to apoptosis in the human cervical carcinoma cell line HeLa [88]. The prevention of the interaction between NR4A1 and Bcl-B leads to an increase in cell viability in the human colon cancer cell line HCT-116 and the inhibition of autophagy in the human brain neuroglioma cell line H4 [89].

Celastrol, a triterpene extracted from the Chinese “Thunder of God Vine,” binds to the LBD of NR4A1 [90]. Tumor necrosis factor-α induces the translocation of tumor necrosis factor receptor-associated factor 2, which is an E3 ubiquitin ligase [91, 92], to mitochondria, and celastrol induces NR4A1 translocation to mitochondria by interacting with tumor necrosis factor receptor-associated factor 2. This leads to the recruitment of p62 via ubiquitination of NR4A1 at mitochondria, followed by binding of p62 to LC3 and induction of mitophagy in the human hepatoblastoma cell line HepG2 [90]. p62 associates with ubiquitinated target proteins via its ubiquitin-associated region and LC3 via its LC3-interacting region to isolate ubiquitinated target proteins, leading to autophagic degradation [93, 94]. The N-terminal intrinsically disordered region of NR4A1 associates with the N-terminal Phox and Bem1 region of p62, and the ubiquitinated LBD of NR4A1 associates with the ubiquitin-associated region of p62. The N-terminal intrinsically disordered region of NR4A alone loses its ability to bind to and isolate mitochondria. In contrast, although the ubiquitinated LBD of NR4A1 alone can isolate mitochondria, it loses the ability to conduct mitophagy [95].

Fenretinide [N-(4-hydroxyphenyl)retinamide, also called 4-HPR] is a synthetic analog of all-trans-retinoic acid and induces NR4A1 expression and translocation from the nucleus to mitochondria. Furthermore, apoptosis is induced by fenretinide, and NR4A1 knockdown partially suppresses this fenretinide-induced apoptosis [96]. In contrast, XS561, a synthetic small molecule, binds to the LBD of NR4A1 and induces NR4A1 translocation to mitochondria and the association between NR4A1 and Bcl-2. In turn, XS561 induces apoptosis dependent on NR4A1 [97]. 12-O-tetradecanoylphorbol 13-acetate induces the formation of a heterodimer of NR4A1 with RXRα (NR4A1/RXRα heterodimer), leading to the translocation of NR4A1/RXRα heterodimer to mitochondria in the human prostate adenocarcinoma cell line LNCaP. The DBDs of NR4A1 and RXRα are required for heterodimer formation, and the NES of RXRα is important for the export of the NR4A1/RXRα heterodimer from the nucleus. Moreover, translocation of the NR4A1/RXRα heterodimer might depend on the association of Bcl-2 and NR4A1 in mitochondria. 9-cis-retinoic acid and SR11237, which are RXR agonists, inhibit NR4A1/RXRα heterodimer translocation to mitochondria and apoptosis induced by tetradecanoylphorbol 13-acetate or CD437, which is a synthetic retinoid that is an RARγ-selective agonist and also known as 6-[3-(1-adamantyl)-4-hydroxyphenyl]-2 naphthalene carboxylic acid [98]. In contrast, 9-cis-retinoic acid induces NR4A1/RXRα heterodimer formation and NR4A1 and RXRα translocation to mitochondria in human cervical carcinoma cell line HeLa [99]. These conflicting results may be attributed to various factors stemming from cell type differences. Insulin-like growth factor (IGF)-binding protein-3 (IGFBP-3) induces apoptosis independently of IGF binding [100]. Translocation of NR4A1 from the nucleus to mitochondria in human prostate carcinoma cell line 22Rv1, mouse IGF receptor-negative embryonic fibroblasts, and rat chondrocytes is induced by treatment with IGFBP-3 [101, 102]. RXRα also translocates to mitochondria by IGFBP-3. RXRα is necessary for NR4A1 nuclear export and the activation of caspase-3 and caspase-7 upon treatment of IGFBP-3 [101]. In addition, IGFBP-3 associates with NR4A1 in the cytoplasm, not the nucleus. NR4A1 is involved in the activation of caspase-3 and caspase-7 upon IGFBP-3 treatment in mouse embryonic fibroblast cells [103].

### 4.2. Effect of NR4A1 on Apoptosis and ER Stress in the ER

NR4A1 can localize to the ER by interacting with Bcl-2 or translocon-associated protein subunit γ (TRAPγ) (Figure 4). TRAPγ, also known as signal sequence receptor subunit 3, is located in the ER and binds to the LBD of NR4A1. Tetradecanoylphorbol 13-acetate or CD437 induces the binding between NR4A1 and TRAPγ, leading to NR4A1 translocation to the ER. TRAPγ forms the TRAP complex, consisting of α, β, γ, and δ subunits, and the TRAP complex is located in the ER membrane, indicating NR4A1 is localized in the ER membrane. Furthermore, apoptosis induced by tunicamycin, the ER stress inducer, with tetradecanoylphorbol 13-acetate or CD437 is dependent on NR4A1 [104]. CD437 induces NR4A1 translocation from the nucleus to the ER. NR4A1 overlaps with protein disulfide isomerase, which is found in the membrane of the ER, indicating that NR4A1 is present at the membrane of the ER. Furthermore, NR4A1 interacts with Bcl-2, leading to the release of calcium ions from the ER and apoptosis in the human neuroblastoma cell line SK-N-SH and the human esophageal squamous carcinoma cell lines EC109 and EC9706 [105]. A 4-(quinoline-4-amino)benzoylhydrazide (4-PQBH) derivative induces NR4A1 expression, ER stress, vacuolation, and autophagy. 4-PQBH binds to NR4A1 and induces NR4A1 translocation to the ER. ER stress and vacuolation induced by 4-PQBH depend on NR4A1 [106]. In addition, cigarette smoke extract induces ER stress and increases NR4A1 expression. Cigarette smoke extract also induces NR4A1 translocation to the ER, and NR4A1 is involved in ER stress induced by cigarette smoke extract. NR4A1 is colocalized with ER-Tracker Green, which contains glibenclamide that binds to sulphonylurea receptors of ATP-sensitive K^+^ channels prominently expressed in the ER membrane [107].

## 5. Concluding Remarks

Nuclear receptors have a profound effect on physiological functions because they control the expression of numerous genes in a variety of cells. NR4A1 is a nuclear receptor belonging to the NR4A subfamily of the nuclear receptor superfamily. NR4A1 expression is induced by various stress factors, and NR4A1is involved in the regulation of cell growth, apoptosis, the immune system, and various cancers. Therefore, NR4A1 is closely linked to many diseases (Table 2). NR4A1 is retained in the nucleus through its interaction with T-cell lymphoma invasion and metastasis-inducing protein 1, preventing Bcl-2 activation and contributing to lung cancer cell survival [83]. Translocation of NR4A1 to mitochondria is related to the apoptosis of liver cancer [108, 109] and leukemia cells [110]. NR4A1 is associated with suppression of immune responses, as it is involved in the differentiation of T regulatory cells that secrete inhibitory cytokines such as IL-10, IL-35, and transforming growth factor-β [111]. NR4A1 is involved in regulating extracellular matrix, tolerance, and inhibition of phagocytosis in bone marrow-derived macrophages, indicating the protective effect of NR4A1 against multiple chronic inflammatory joint diseases such as rheumatoid arthritis, which are affected by macrophages [112]. Furthermore, NR4A1 inhibits inflammation and matrix metalloproteinases' expression in chondrocytes and attenuates osteoarthritis [113]. NRA41 plays a key role in promoting apoptosis and inhibiting the secretion of IL-1 and IL-6 in the fibroblast-like synovial cell line MH7A. Furthermore, NR4A1 expression is associated with attenuated inflammatory cell infiltration and synovial hypertrophy in collagen-induced arthritis rats, suggesting a protective role for NR4A1 in the progression of rheumatoid arthritis [114, 115]. In mice injected with α-synuclein preformed fibrils, a widely used model of Parkinson's disease, NR4A1 translocates to mitochondria and reduces the loss of dopamine neurons [116]. In addition, deficiency of NR4A1 increases nigra striatum damage [117]. Thus, NR4A1 might have a protective role in Parkinson's disease. Knockout of NR4A1 displays earlier onset and more severe clinical experimental autoimmune encephalomyelitis, which is related to multiple sclerosis [118]. In addition, knockout of NR4A1 increases in the number of infiltrating myeloid cells and activated microglia during the early prodromal phase of experimental autoimmune encephalomyelitis [119].

In the nucleus, NR4A1 directly binds to specific sequences, such as NBRE, Nur-responsive element, and direct repeat 5, present in genomic DNA to control gene expression, and indirectly controls gene expression by binding to other transcription factors, such as SP-1. In contrast, NR4A1 can translocate out of the nucleus and interacts with other proteins in the cytoplasm to exert diverse functions. NR4A1 binds to LPS and mtDNA in the cytoplasm, leading to binding to NLRP3 and induction of inflammasome formation. Furthermore, NR4A1 can translocate to mitochondria and the ER, and participates in autophagy and apoptosis induction. Therefore, NR4A1 exerts different functions depending on its localization in the cell. Although the endogenous ligand for NR4A1 has not been identified, various structurally distinct compounds that bind to NR4A1 have been identified [120]. These compounds are those that act as agonists to activate and antagonists to inactivate NR4A1 and those that induce the nuclear export of NR4A1. Because NR4A1 is involved in diseases such as cancer, NR4A1 may be an important pharmacological target. Therefore, detailed functional analysis of NR4A1 expression, transcriptional activity, and localization and the development of drugs that alter NR4A1 function will be useful in developing treatments for diseases such as cancer.

## Figures and Tables

**Table 1:** The direct and indirect influences of NR4A1 on gene expression.

Direct or indirect influences of NR4A1	Influences of NR4A1 on gene expression
Direct influences of NR4A1	Regulation of gene expression via binding to NGFI-B response element (AAAGGTCA)
	Regulation of gene expression via binding to Nur-responsive element (TGATATTTacctccAAATGCCA and TGACCTTTacctccAAAGGTCA)
	Regulation of gene expression via binding to direct repeat 5 (AGGTCAnnnnnAGGTCA)

Indirect influences of NR4A1	Reduction of the transcriptional activity of SP1 and SP4
	Reduction of the transcriptional activity of Fos, FosB, and Jun
	Reduction of the transcriptional activity of RNA polymerase II
	Reduction of the transcriptional activity of NF-κB
	Reduction of the transcriptional activity of AP-1
	Reduction of the transcriptional activity of p53

**Table 2:** NR4A1-related diseases and the role of NR4A1.

NR4A1-related diseases		Roles of NR4A1
Cancer	Lung cancer	Cancer cell survival
	Liver cancer	Induction of apoptosis
	Leukemia	Induction of apoptosis

Immune system	Immune responses	Suppression
	Rheumatoid arthritis	Protective role against the progression

Neurodegenerative diseases	Parkinson's disease	Protective role against the progression
	Multiple sclerosis	Protective role against earlier onset and more severe clinical experimental autoimmune encephalomyelitis

## Data Availability

Data is sharing not applicable to this article as no datasets were generated or analyzed during the current study.

## References

[1] Weikum E. R., Liu X., Ortlund E. A. (2018). The Nuclear Receptor Superfamily: A Structural Perspective. *Protein Science*.

[2] Shiota M., Fujimoto N., Kashiwagi E., Eto M. (2019). The Role of Nuclear Receptors in Prostate Cancer. *Cells*.

[3] Porter B. A., Ortiz M. A., Bratslavsky G., Kotula L. (2019). Structure and Function of the Nuclear Receptor Superfamily and Current Targeted Therapies of Prostate Cancer. *Cancers (Basel)*.

[4] Pan P., Chen X. (2020). Nuclear Receptors as Potential Therapeutic Targets for Myeloid Leukemia. *Cells*.

[5] Hazel T. G., Nathans D., Lau L. F. (1988). A Gene Inducible by Serum Growth Factors Encodes a Member of the Steroid and Thyroid Hormone Receptor Superfamily. *Proceedings of the National Academy of Sciences of the USA*.

[6] Milbrandt J. (1988). Nerve Growth Factor Induces a Gene Homologous to the Glucocorticoid Receptor Gene. *Neuron*.

[7] Chawnshang C., Kokontis J., Shutsung L., Yijan C. (1989). Isolation and Characterization of Human TR3 Receptor: A Member of Steroid Receptor Superfamily. *Journal of Steroid Biochemistry*.

[8] Li Q. X., Ke N., Sundaram R., Wong-Staal F. (2006). NR4A1, 2, 3--An Orphan Nuclear Hormone Receptor Family Involved in Cell Apoptosis and Carcinogenesis. *Histology & Histopathology*.

[9] Liu X., Wang Y., Lu H. (2019). Genome-wide Analysis Identifies NR4A1 as a Key Mediator of T Cell Dysfunction. *Nature*.

[10] Odagiu L., May J., Boulet S., Baldwin T. A., Labrecque N. (2020). Role of the Orphan Nuclear Receptor NR4A Family in T-Cell Biology. *Frontiers in Endocrinology*.

[11] Deng S., Chen B., Huo J., Liu X. (2022). Therapeutic Potential of NR4A1 in Cancer: Focus on Metabolism. *Frontiers in Oncology*.

[12] Herring J. A., Elison W. S., Tessem J. S. (2019). Function of Nr4a Orphan Nuclear Receptors in Proliferation, Apoptosis and Fuel Utilization Across Tissues. *Cells*.

[13] Upadhyay S., Hailemariam A. E., Mariyam F. (2024). Bis-Indole Derivatives as Dual Nuclear Receptor 4A1 (NR4A1) and NR4A2 Ligands. *Biomolecules*.

[14] Yin C., Liu H., Shan Y. (2019). Cytosporone B as a Biological Preservative: Purification, Fungicidal Activity and Mechanism of Action Against *Geotrichum citri-aurantii*. *Biomolecules*.

[15] Zhan Y., Du X., Chen H. (2008). Cytosporone B Is an Agonist for Nuclear Orphan Receptor Nur77. *Nature Chemical Biology*.

[16] Shrestha R., Mohankumar K., Martin G. (2021). Flavonoids Kaempferol and Quercetin Are Nuclear Receptor 4A1 (NR4A1, Nur77) Ligands and Inhibit Rhabdomyosarcoma Cell and Tumor Growth. *Journal of Experimental & Clinical Cancer Research*.

[17] Zhang L., Martin G., Mohankumar K., Hampton J. T., Liu W. R., Safe S. (2022). Resveratrol Binds Nuclear Receptor 4a1 (Nr4a1) and Acts as an Nr4a1 Antagonist in Lung Cancer Cells. *Molecular Pharmacology*.

[18] Zhang L., Martin G., Mohankumar K., Wright G. A., Mariyam F., Safe S. (2023). Piperlongumine Is a Ligand for the Orphan Nuclear Receptor 4A1 (NR4A1). *Frontiers in Pharmacology*.

[19] Willems S., Kilu W., Ni X. (2020). The Orphan Nuclear Receptor Nurr1 Is Responsive to Non-Steroidal Anti-Inflammatory Drugs. *Communications Chemistry*.

[20] Lee M., Upadhyay S., Mariyam F. (2023). Flavone and Hydroxyflavones Are Ligands That Bind the Orphan Nuclear Receptor 4A1 (NR4A1). *International Journal of Molecular Sciences*.

[21] Vinayavekhin N., Saghatelian A. (2011). Discovery of a Protein-Metabolite Interaction Between Unsaturated Fatty Acids and the Nuclear Receptor Nur77 Using a Metabolomics Approach. *Journal of the American Chemical Society*.

[22] Maxwell M. A., Muscat G. E. (2006). The NR4A Subgroup: Immediate Early Response Genes With Pleiotropic Physiological Roles. *Nuclear Receptor Signaling*.

[23] Katagiri Y., Takeda K., Yu Z. X., J Ferrans V., Ozato K., Guroff G. (2000). Modulation of Retinoid Signalling Through NGF-Induced Nuclear Export of NGFI-B. *Nature Cell Biology*.

[24] Aggarwal A., Agrawal D. K. (2014). Importins and Exportins Regulating Allergic Immune Responses. *Mediators of Inflammation*.

[25] Lu J., Wu T., Zhang B. (2021). Types of Nuclear Localization Signals and Mechanisms of Protein Import Into the Nucleus. *Cell Communication and Signaling*.

[26] Sun Z., Cao X., Jiang M. M. (2012). Inhibition of Beta-Catenin Signaling by Nongenomic Action of Orphan Nuclear Receptor Nur77. *Oncogene*.

[27] Papac-Milicevic N., Breuss J. M., Zaujec J. (2012). The Interferon Stimulated Gene 12 Inactivates Vasculoprotective Functions of NR4A Nuclear Receptors. *Circulation Research*.

[28] Pekarsky Y., Hallas C., Palamarchuk A. (2001). Akt Phosphorylates and Regulates the Orphan Nuclear Receptor Nur77. *Proceedings of the National Academy of Sciences of the USA*.

[29] Zhou Y., Zhao W., Xie G. (2014). Induction of Nur77-Dependent Apoptotic Pathway by a Coumarin Derivative Through Activation of JNK and p38 MAPK. *Carcinogenesis*.

[30] Chang L. F., Lin P. C., Ho L. I. (2011). Overexpression of the Orphan Receptor Nur77 and Its Translocation Induced by PCH4 May Inhibit Malignant Glioma Cell Growth and Induce Cell Apoptosis. *Journal of Surgical Oncology*.

[31] Liu P. Y., Sheu J. J., Lin P. C. (2012). Expression of Nur77 Induced by an N-Butylidenephthalide Derivative Promotes Apoptosis and Inhibits Cell Growth in Oral Squamous Cell Carcinoma. *Investigational New Drugs*.

[32] Wang A., Rud J., Olson C. M., Anguita J., Osborne B. A. (2009). Phosphorylation of Nur77 by the MEK-ERK-RSK Cascade Induces Mitochondrial Translocation and Apoptosis in T Cells. *The Journal of Immunology*.

[33] Han Y. H., Cao X., Lin B. (2006). Regulation of Nur77 Nuclear Export by c-Jun N-Terminal Kinase and Akt. *Oncogene*.

[34] Thompson J., Burger M. L., Whang H., Winoto A. (2010). Protein Kinase C Regulates Mitochondrial Targeting of Nur77 and Its Family Member Nor-1 in Thymocytes Undergoing Apoptosis. *European Journal of Immunology*.

[35] Li X. X., Wang Z. J., Zheng Y. (2018). Nuclear Receptor Nur77 Facilitates Melanoma Cell Survival Under Metabolic Stress by Protecting Fatty Acid Oxidation. *Molecular Cell*.

[36] Zarraga-Granados G., Mucino-Hernandez G., Sanchez-Carbente M. R. (2020). The Nuclear Receptor NR4A1 Is Regulated by SUMO Modification to Induce Autophagic Cell Death. *PLoS One*.

[37] Zhang L., Xie F., Zhang J., Dijke P. T., Zhou F. (2017). SUMO-Triggered Ubiquitination of NR4A1 Controls Macrophage Cell Death. *Cell Death & Differentiation*.

[38] Dodat F., Mader S., Levesque D. (2021). Minireview: What Is Known About SUMOylation Among NR4A Family Members?. *Journal of Molecular Biology*.

[39] Wilson T. E., Fahrner T. J., Johnston M., Milbrandt J. (1991). Identification of the DNA Binding Site for NGFI-B by Genetic Selection in Yeast. *Science*.

[40] Maira M., Martens C., Philips A., Drouin J. (1999). Heterodimerization Between Members of the Nur Subfamily of Orphan Nuclear Receptors as a Novel Mechanism for Gene Activation. *Molecular and Cellular Biology*.

[41] Philips A., Lesage S., Gingras R. (1997). Novel Dimeric Nur77 Signaling Mechanism in Endocrine and Lymphoid Cells. *Molecular and Cellular Biology*.

[42] Zetterstrom R. H., Solomin L., Mitsiadis T., Olson L., Perlmann T. (1996). Retinoid X Receptor Heterodimerization and Developmental Expression Distinguish the Orphan Nuclear Receptors NGFI-B, Nurr1, and Nor1. *Molecular Endocrinology*.

[43] Perlmann T., Jansson L. (1995). A Novel Pathway for Vitamin A Signaling Mediated by RXR Heterodimerization With NGFI-B and NURR1. *Genes & Development*.

[44] Lee S. O., Chintharlapalli S., Liu S. (2009). p21 Expression Is Induced by Activation of Nuclear Nerve Growth Factor-Induced Bα (Nur77) in Pancreatic Cancer Cells. *Molecular Cancer Research*.

[45] Yerra V. G., Drosatos K. (2023). Specificity Proteins (SP) and Kruppel-Like Factors (KLF) in Liver Physiology and Pathology. *International Journal of Molecular Sciences*.

[46] Lee S. O., Abdelrahim M., Yoon K. (2010). Inactivation of the Orphan Nuclear Receptor TR3/Nur77 Inhibits Pancreatic Cancer Cell and Tumor Growth. *Cancer Research*.

[47] Hedrick E., Lee S. O., Doddapaneni R., Singh M., Safe S. (2016). NR4A1 Antagonists Inhibit β1-Integrin-Dependent Breast Cancer Cell Migration. *Molecular and Cellular Biology*.

[48] Karki K., Wright G. A., Mohankumar K., Jin U. H., Zhang X. H., Safe S. (2020). A Bis-Indole-Derived NR4A1 Antagonist Induces PD-L1 Degradation and Enhances Antitumor Immunity. *Cancer Research*.

[49] Guo H., Golczer G., Wittner B. S. (2021). NR4A1 Regulates Expression of Immediate Early Genes, Suppressing Replication Stress in Cancer. *Molecular Cell*.

[50] Li L., Liu Y., Chen H. Z. (2015). Impeding the Interaction Between Nur77 and P38 Reduces LPS-Induced Inflammation. *Nature Chemical Biology*.

[51] Yang P. B., Hou P. P., Liu F. Y. (2020). Blocking PPARγ Interaction Facilitates Nur77 Interdiction of Fatty Acid Uptake and Suppresses Breast Cancer Progression. *Proceedings of the National Academy of Sciences of the USA*.

[52] Zhang L., Afolabi L. O., Wan X., Li Y., Chen L. (2020). Emerging Roles of Tripartite Motif-Containing Family Proteins (TRIMs) in Eliminating Misfolded Proteins. *Frontiers in Cell and Developmental Biology*.

[53] Zhao B. X., Chen H. Z., Lei N. Z. (2006). p53 Mediates the Negative Regulation of MDM2 by Orphan Receptor TR3. *The EMBO Journal*.

[54] Zhou Y., Nakajima R., Shirasawa M. (2023). Expanding Roles of the E2F-RB-P53 Pathway in Tumor Suppression. *Biology*.

[55] Kubbutat M. H., Jones S. N., Vousden K. H. (1997). Regulation of P53 Stability by Mdm2. *Nature*.

[56] Klein A. M., de Queiroz R. M., Venkatesh D., Prives C. (2021). The Roles and Regulation of MDM2 and MDMX: It Is Not Just About P53. *Genes & Development*.

[57] Chen X., Xu X., Chen Y. (2021). Structure of an Activated DNA-PK and Its Implications for NHEJ. *Molecular Cell*.

[58] Zhao B. X., Chen H. Z., Du X. D. (2011). Orphan Receptor TR3 Enhances P53 Transactivation and Represses DNA Double-Strand Break Repair in Hepatoma Cells Under Ionizing Radiation. *Molecular Endocrinology*.

[59] Xiong X., Lai X., Li A., Liu Z., Ma N. (2021). Diversity Roles of CHD1L in Normal Cell Function and Tumorigenesis. *Biomarker research*.

[60] Chen L., Hu L., Chan T. H. (2009). Chromodomain Helicase/Adenosine Triphosphatase DNA Binding Protein 1-Like (CHD1l) Gene Suppresses the Nucleus-to-Mitochondria Translocation of Nur77 to Sustain Hepatocellular Carcinoma Cell Survival. *Hepatology*.

[61] Yu F., Yu C., Li F. (2021). Wnt/β-Catenin Signaling in Cancers and Targeted Therapies. *Signal Transduction and Targeted Therapy*.

[62] Wang B., Li X., Liu L., Wang M. (2020). β-Catenin: Oncogenic Role and Therapeutic Target in Cervical Cancer. *Biological Research*.

[63] Shimada K., Ishii N., Nambara T. (1986). Occurrence of Bufadienolides in the Skin of *Bufo viridis* Laur. *Chemical & Pharmaceutical Bulletin*.

[64] Lu W. J., Chua M. S., Wei W., So S. K. (2015). NDRG1 Promotes Growth of Hepatocellular Carcinoma Cells by Directly Interacting With GSK-3β and Nur77 to Prevent β-Catenin Degradation. *Oncotarget*.

[65] Zhu F., Ma J., Li W. (2023). The Orphan Receptor Nur77 Binds Cytoplasmic LPS to Activate the Non-Canonical NLRP3 Inflammasome. *Immunity*.

[66] Maelfait J., Vercammen E., Janssens S. (2008). Stimulation of Toll-Like Receptor 3 and 4 Induces Interleukin-1β Maturation by Caspase-8. *Journal of Experimental Medicine*.

[67] Jin M. S., Lee J. O. (2008). Structures of the Toll-Like Receptor Family and Its Ligand Complexes. *Immunity*.

[68] Akbal A., Dernst A., Lovotti M., Mangan M. S. J., McManus R. M., Latz E. (2022). How Location and Cellular Signaling Combine to Activate the NLRP3 Inflammasome. *Cellular and Molecular Immunology*.

[69] Chai R., Li Y., Shui L., Ni L., Zhang A. (2023). The Role of Pyroptosis in Inflammatory Diseases. *Frontiers in Cell and Developmental Biology*.

[70] Moretti J., Blander J. M. (2021). Increasing Complexity of NLRP3 Inflammasome Regulation. *Journal of Leukocyte Biology*.

[71] Weindel C. G., Ellzey L. M., Martinez E. L., Watson R. O., Patrick K. L. (2023). Gasdermins Gone Wild: New Roles for GSDMs in Regulating Cellular Homeostasis. *Trends in Cell Biology*.

[72] Miao R., Jiang C., Chang W. Y. (2023). Gasdermin D Permeabilization of Mitochondrial Inner and Outer Membranes Accelerates and Enhances Pyroptosis. *Immunity*.

[73] Huang L. S., Hong Z., Wu W. (2020). mtDNA Activates cGAS Signaling and Suppresses the YAP-Mediated Endothelial Cell Proliferation Program to Promote Inflammatory Injury. *Immunity*.

[74] Wang W. J., Wang Y., Chen H. Z. (2014). Orphan Nuclear Receptor TR3 Acts in Autophagic Cell Death via Mitochondrial Signaling Pathway. *Nature Chemical Biology*.

[75] Li Y., Zheng W., Lu Y. (2021). BNIP3L/NIX-Mediated Mitophagy: Molecular Mechanisms and Implications for Human Disease. *Cell Death & Disease*.

[76] Kiriyama Y., Nochi H. (2017). Intra- and Intercellular Quality Control Mechanisms of Mitochondria. *Cells*.

[77] Wang W., Chen X., Zhang L. (2020). Atomic Structure of Human TOM Core Complex. *Cell Discovery*.

[78] Sayyed U. M. H., Mahalakshmi R. (2022). Mitochondrial Protein Translocation Machinery: From TOM Structural Biogenesis to Functional Regulation. *Journal of Biological Chemistry*.

[79] Lin B., Kolluri S. K., Lin F. (2004). Conversion of Bcl-2 From Protector to Killer by Interaction With Nuclear Orphan Receptor Nur77/TR3. *Cell*.

[80] Kapoor I., Bodo J., Hill B. T., Hsi E. D., Almasan A. (2020). Targeting BCL-2 in B-Cell Malignancies and Overcoming Therapeutic Resistance. *Cell Death & Disease*.

[81] Kolluri S. K., Zhu X., Zhou X. (2008). A Short Nur77-Derived Peptide Converts Bcl-2 From a Protector to a Killer. *Cancer Cell*.

[82] Chen X., Cao X., Tu X. (2019). BI1071, A Novel Nur77 Modulator, Induces Apoptosis of Cancer Cells by Activating the Nur77-Bcl-2 Apoptotic Pathway. *Molecular Cancer Therapeutics*.

[83] Payapilly A., Guilbert R., Descamps T. (2021). TIAM1-RAC1 Promote Small-Cell Lung Cancer Cell Survival Through Antagonizing Nur77-Induced BCL2 Conformational Change. *Cell Reports*.

[84] Banjara S., Suraweera C. D., Hinds M. G., Kvansakul M. (2020). The Bcl-2 Family: Ancient Origins, Conserved Structures, and Divergent Mechanisms. *Biomolecules*.

[85] Ke N., Godzik A., Reed J. C. (2001). Bcl-B, A Novel Bcl-2 Family Member That Differentially Binds and Regulates Bax and Bak. *Journal of Biological Chemistry*.

[86] Opferman J. T., Kothari A. (2018). Anti-Apoptotic BCL-2 Family Members in Development. *Cell Death & Differentiation*.

[87] Pervushin N. V., Kopeina G. S., Zhivotovsky B. (2023). Bcl-B: An “Unknown” Protein of the Bcl-2 Family. *Biology Direct*.

[88] Luciano F., Krajewska M., Ortiz-Rubio P. (2007). Nur77 Converts Phenotype of Bcl-B, An Antiapoptotic Protein Expressed in Plasma Cells and Myeloma. *Blood*.

[89] Godoi P. H. C., Wilkie-Grantham R. P., Hishiki A. (2016). Orphan Nuclear Receptor NR4A1 Binds a Novel Protein Interaction Site on Anti-Apoptotic B Cell Lymphoma Gene 2 Family Proteins. *Journal of Biological Chemistry*.

[90] Hu M., Luo Q., Alitongbieke G. (2017). Celastrol-Induced Nur77 Interaction With TRAF2 Alleviates Inflammation by Promoting Mitochondrial Ubiquitination and Autophagy. *Molecular Cell*.

[91] Siegmund D., Wagner J., Wajant H. (2022). TNF Receptor Associated Factor 2 (TRAF2) Signaling in Cancer. *Cancers (Basel)*.

[92] Dhillon B., Aleithan F., Abdul-Sater Z., Abdul-Sater A. A. (2019). The Evolving Role of TRAFs in Mediating Inflammatory Responses. *Frontiers in Immunology*.

[93] Kumar A. V., Mills J., Lapierre L. R. (2022). Selective Autophagy Receptor p62/SQSTM1, A Pivotal Player in Stress and Aging. *Frontiers in Cell and Developmental Biology*.

[94] Berkamp S., Mostafavi S., Sachse C. (2021). Structure and Function of p62/SQSTM1 in the Emerging Framework of Phase Separation. *FEBS Journal*.

[95] Peng S. Z., Chen X. H., Chen S. J. (2021). Phase Separation of Nur77 Mediates Celastrol-Induced Mitophagy by Promoting the Liquidity of p62/SQSTM1 Condensates. *Nature Communications*.

[96] Xiong J., Kuang X., Lu T. (2019). Fenretinide-Induced Apoptosis of Acute Myeloid Leukemia Cells via NR4A1 Translocation Into Mitochondria and Bcl-2 Transformation. *Journal of Cancer*.

[97] Chen X., Gao M., Xia Y. (2024). Phase Separation of Nur77 Mediates XS561-Induced Apoptosis by Promoting the Formation of Nur77/Bcl-2 Condensates. *Acta Pharmaceutica Sinica B*.

[98] Cao X., Liu W., Lin F. (2004). Retinoid X Receptor Regulates Nur77/Thyroid Hormone Receptor 3-Dependent Apoptosis by Modulating Its Nuclear Export and Mitochondrial Targeting. *Molecular and Cellular Biology*.

[99] Zhao W. X., Tian M., Zhao B. X. (2007). Orphan Receptor TR3 Attenuates the p300-Induced Acetylation of Retinoid X Receptor-Alpha. *Molecular Endocrinology*.

[100] Varma Shrivastav S., Bhardwaj A., Pathak K. A., Shrivastav A. (2020). Insulin-Like Growth Factor Binding Protein-3 (IGFBP-3): Unraveling the Role in Mediating IGF-independent Effects Within the Cell. *Frontiers in Cell and Developmental Biology*.

[101] Lee K. W., Ma L., Yan X., Liu B., Zhang X. K., Cohen P. (2005). Rapid Apoptosis Induction by IGFBP-3 Involves an Insulin-Like Growth Factor-Independent Nucleomitochondrial Translocation of RXRα/Nur77. *Journal of Biological Chemistry*.

[102] Wei Z., Li H. H. (2015). IGFBP-3 May Trigger Osteoarthritis by Inducing Apoptosis of Chondrocytes Through Nur77 Translocation. *International Journal of Clinical and Experimental Pathology*.

[103] Lee K.-W., Cobb L. J., Paharkova-Vatchkova V., Liu B., Milbrandt J., Cohen P. (2007). Contribution of the Orphan Nuclear Receptor Nur77 to the Apoptotic Action of IGFBP-3. *Carcinogenesis*.

[104] Chen H. Z., Wen Q., Wang W. J., He J. P., Wu Q. (2013). The Orphan Nuclear Receptor TR3/Nur77 Regulates ER Stress and Induces Apoptosis via Interaction With TRAPγ. *The International Journal of Biochemistry & Cell Biology*.

[105] Liang B., Song X., Liu G. (2007). Involvement of TR3/Nur77 Translocation to the Endoplasmic Reticulum in ER Stress-Induced Apoptosis. *Experimental Cell Research*.

[106] Li B., Huang J., Liu J. (2022). Discovery of a Nur77-Mediated Cytoplasmic Vacuolation and Paraptosis Inducer (4-PQBH) for the Treatment of Hepatocellular Carcinoma. *Bioorganic Chemistry*.

[107] Chang C., He F., Ao M. (2023). Inhibition of Nur77 Expression and Translocation by Compound B6 Reduces ER Stress and Alleviates Cigarette Smoke-Induced Inflammation and Injury in Bronchial Epithelial Cells. *Frontiers in Pharmacology*.

[108] Yang H., Nie Y., Li Y., Wan Y. J. (2011). ERK1/2 Deactivation Enhances Cytoplasmic Nur77 Expression Level and Improves the Apoptotic Effect of Fenretinide in Human Liver Cancer Cells. *Biochemical Pharmacology*.

[109] Li X., Chen Q., Liu J. (2022). Orphan Nuclear Receptor Nur77 Mediates the Lethal Endoplasmic Reticulum Stress and Therapeutic Efficacy of Cryptomeridiol in Hepatocellular Carcinoma. *Cells*.

[110] Wang C., He H., Dou G. (2017). Ginsenoside 20(S)-Rh2 Induces Apoptosis and Differentiation of Acute Myeloid Leukemia Cells: Role of Orphan Nuclear Receptor Nur77. *Journal of Agricultural and Food Chemistry*.

[111] Li W., Hang S., Fang Y. (2021). A Bacterial Bile Acid Metabolite Modulates T_reg_ Activity Through the Nuclear Hormone Receptor NR4A1. *Cell Host & Microbe*.

[112] Hamers A. A., Argmann C., Moerland P. D. (2016). Nur77-Deficiency in Bone Marrow-Derived Macrophages Modulates Inflammatory Responses, Extracellular Matrix Homeostasis, Phagocytosis and Tolerance. *BMC Genomics*.

[113] Xiong Y., Ran J., Xu L. (2020). Reactivation of NR4A1 Restrains Chondrocyte Inflammation and Ameliorates Osteoarthritis in Rats. *Frontiers in Cell and Developmental Biology*.

[114] Song H., Wu H., Dong J., Huang S., Ye J., Liu R. (2021). Ellagic Acid Alleviates Rheumatoid Arthritis in Rats Through Inhibiting MTA1/HDAC1-Mediated Nur77 Deacetylation. *Mediators of Inflammation*.

[115] De Silva S., Han S., Zhang X., Huston D. P., Winoto A., Zheng B. (2005). Reduction of the Incidence and Severity of Collagen-Induced Arthritis by Constitutive Nur77 Expression in the T Cell Lineage. *Arthritis & Rheumatism*.

[116] Yin S., Shen M., Zhang Y. (2024). Nur77 Increases Mitophagy and Decreases Aggregation of Alpha-Synuclein by Modulating the p-c-Abl/p-PHB2 Y121 in Alpha-Synuclein PFF SH-SY5Y Cells and Mice. *European Journal of Medicinal Chemistry*.

[117] Mount M. P., Zhang Y., Amini M. (2013). Perturbation of Transcription Factor Nur77 Expression Mediated by Myocyte Enhancer Factor 2D (MEF2D) Regulates Dopaminergic Neuron Loss in Response to 1-Methyl-4-Phenyl-1,2,3,6-Tetrahydropyridine (MPTP). *Journal of Biological Chemistry*.

[118] Wang L. M., Zhang Y., Li X. (2018). Nr4a1 Plays a Crucial Modulatory Role in Th1/Th17 Cell Responses and CNS Autoimmunity. *Brain, Behavior, and Immunity*.

[119] Rothe T., Ipseiz N., Faas M. (2017). The Nuclear Receptor Nr4a1 Acts as a Microglia Rheostat and Serves as a Therapeutic Target in Autoimmune-Driven Central Nervous System Inflammation. *The Journal of Immunology*.

[120] Qin J., Niu B., Chen X. (2023). Discovery of 5-(Pyrimidin-2-Ylamino)-1H-Indole-2-Carboxamide Derivatives as Nur77 Modulators With Selective and Potent Activity Against Triple-Negative Breast Cancer. *Journal of Medicinal Chemistry*.

